# Neuroprotective and reparative effects of endoplasmic reticulum luminal proteins – mesencephalic astrocyte-derived neurotrophic factor and cerebral dopamine neurotrophic factor

**DOI:** 10.3325/cmj.2019.60.99

**Published:** 2019-04

**Authors:** Katrina Albert, Mikko Airavaara

**Affiliations:** 1 Institute of Biotechnology, University of Helsinki, Helsinki, Finland; 2 Neuroscience Center, University of Helsinki, Helsinki, Finland

## Abstract

Cerebral dopamine neurotrophic factor (CDNF) and mesencephalic astrocyte-derived neurotrophic factor (MANF) are proteins that have received increasing attention in the last decades. Although they are called neurotrophic factors they are drastically different from neurotrophic factors in their expression and physiological actions. They are located in the lumen of the endoplasmic reticulum (ER) and their basal secretion from neurons is very low. However their secretion is stimulated upon ER calcium depletion by chemical probes such as thapsigargin, a sarco/endoplasmic reticulum Ca^2+^-ATPase (SERCA) pump inhibitor. Exogenous MANF and CDNF possess therapeutic properties in several neurological disease models, including Parkinson’s disease and stroke. Endogenous MANF expression has been shown to be neuroprotective, as well as administration of either CDNF or MANF into the extracellular space. In this review, we focus on their therapeutic effects, regulation of expression and secretion, comparison of their mechanisms of action, and their application to the brain parenchyma as recombinant proteins.

The molecular characterization and protective effects of mesencephalic astrocyte-derived neurotrophic factor (MANF) were first described by Petrova et al (1). The protein was initially characterized from a mesencephalic type-1 astrocyte cell line followed by cloning of MANF from human cDNA. MANF was found to be neuroprotective at ng/mL concentrations on dopaminergic neurons *in vitro*, the results that are difficult to interpret due to the fact that a transmembrane receptor is not identified, as will be discussed below. Our later studies have found that particularly *in vivo*, MANF is neuroprotective when administered as a recombinant protein or delivered via adeno-associated virus (AAV). *In vitro*, we have shown that endogenous MANF is neuroprotective against oxygen-glucose deprivation, when expressed in cells, and when it is localized in the endoplasmic reticulum (ER). However, the neuroprotective effects of recombinant protein added to the culture medium have been variable.

Cerebral dopamine neurotrophic factor (CDNF) was found by using a bioinformatics screen in order to identify proteins with conserved cysteines (2). It was found that CDNF and MANF show homology, and they form a family of proteins with two isoforms in vertebrates and only one isoform in invertebrates. The homology of human CDNF amino acid identity to human MANF is 59%, and 49% and 46% to *D. melanogaster* and *C. elegans* MANF proteins, respectively (2). It was further found that in cultured HEK-293 cells CDNF is secreted after transient transfections. However, the secretion of CDNF from neurons is not as trivial. As discussed below, CDNF was found to be neuroprotective *in vivo*, and found not to have drastic survival-promoting effects when added to the culture medium *in vitro* (2).

Since their discovery, the number of publications with these proteins has increased over the years (Figure 1). There has been great interest due to their success in animal models of Parkinson’s disease and stroke (2,3); and CDNF is currently in clinical trials for Parkinson’s disease. Their properties differ from other neurotrophic factors due to their structure where the N-terminal domain is a saposin-like domain, which is known to interact with lipids, and the C-terminal with its single cysteine bridge and KDEL receptor retention sequence at its end (4). How the structural properties of MANF and CDNF affect their functions, as well as their effects in models of disease and injury, is discussed in greater detail below.

**Figure 1:**
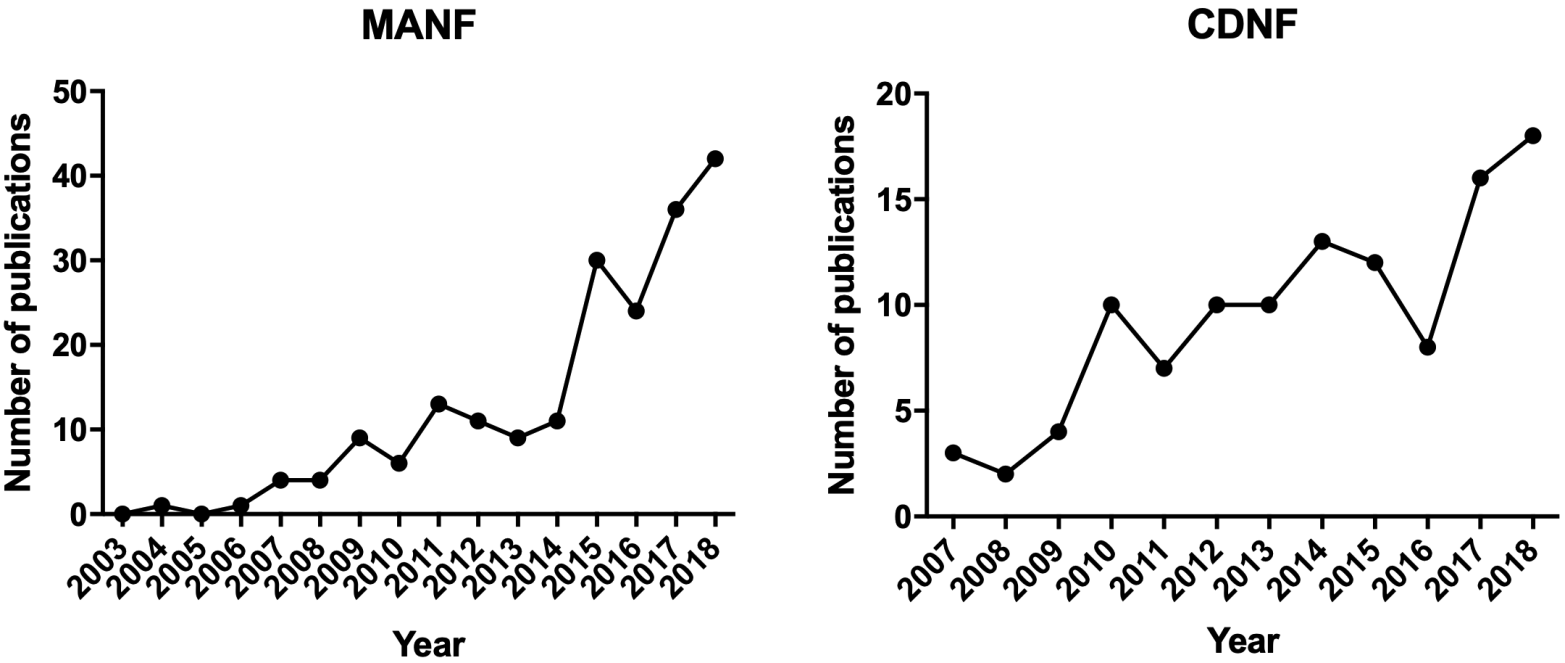
The number of publications on MANF and CDNF since the first paper describing their molecular characterization and therapeutic effects. Source Scopus, March 11, 2019. MANF was searched with keywords Armetl1 OR MANF OR mesencephalic astrocyte-derived neurotrophic factor in the title, abstract, and keywords. CDNF was searched with Armetl1 OR CDNF or cerebral dopamine neurotrophic factor in the title, abstract, and keywords.

## A systematic literature review of MANF and CDNF

The date up to which publications on MANF and CDNF were accessed was March 11, 2019. A search of NCBI’s PubMed with the terms “MANF”, “mesencephalic astrocyte-derived neurotrophic factor”, or “Armetl1”, as well as a search using “CDNF”, “cerebral dopamine neurotrophic factor”, or “Armetl1” (Figure 1) was performed. The authors used an unbiased approach to select the publications, and therefore publications from various laboratories and related to different aspects of the factors were used. Publications indexed in PubMed were chosen since this is considered a reliable and comprehensive resource for searching biomedical literature (5). Though the authors tried to avoid “cherry picking” (6) of published journal articles, it should be noted that the focus of the review was the therapeutic potential of the neurotrophic factors and, therefore, to the best of their ability, all relevant articles are mentioned and referenced. Additionally, the authors aimed to provide a critical appraisal of the literature, also in an unbiased manner.

## Therapeutic effects in Parkinson’s disease

Neurotrophic factors have shown to be both neuroprotective and neurorestorative in animal models of Parkinson’s disease (7). The concept of neurorestoration has been reviewed recently (7), but briefly, it is based on animal and human studies that indicated that at the onset of symptoms, although most striatal dopamine terminals are degenerated, most of the dopaminergic neurons in the substantia nigra pars compacta are still present (8). Furthermore, it has been indicated that when dopamine neurons die they first lose their dopamine phenotype and become metabolically inactive skeletons, enabling neurorestorative therapies to restore the neurotransmitter phenotype and metabolic activity.

Both MANF and CDNF have mainly been used in models of Parkinson’s disease. While MANF has been effective in cells and some animal models, CDNF has shown efficacy in rodent models of the disease, and CDNF is currently in Phase I/II clinical trials for Parkinson’s disease (9).

MANF has shown positive effects in *in vitro* models using 6-hydroxydopamine (6-OHDA), a toxin used to selectively destroy catecholaminergic neurons. Although these are not the neurons specifically affected in Parkinson’s disease patients, they are used to model Parkinson’s disease in cells and animals. It was found that MANF protected against cell death in neuronal cell culture (10,11) However, a study in *C. elegans* demonstrated that the MANF homologue in worms was important for dopamine neurons (12). The results showed that significantly more dopamine neurons were lost over time in the MANF mutants compared to wild-type controls, which was accompanied by ER stress and behavioral deficits. Also, co-expressing MANF and α-synuclein in *C. elegans* protected dopamine neurons, affected behavior, and reduced aggregation of α-synuclein. MANF has also been used in rat models of Parkinson’s disease. When MANF was delivered as a recombinant protein or using AAV unilaterally to the striatum of rats after 6-OHDA, it restored the dopaminergic system, more specifically tyrosine hydroxylase (TH) fibers in the striatum and TH^+^ cells in the substantia nigra pars compacta, as well as rotational behavior (13,14). To complement this, MANF increased stimulus-evoked release of dopamine as well as dopamine turnover in freely moving rats (15). In midbrain slices it potentiated inhibitory GABA current in dopamine neurons (16), demonstrating that MANF has some specific effects on the dopaminergic system. In addition to the AAV-MANF results in 6-OHDA, another study used lentivirus (LV)-MANF, LV-CDNF, as well as a combined vector in the same toxin model. The LV-MANF injection to the substantia nigra had a protective effect on the cells there, and LV-CDNF had a protective effect on the TH^+^ fibers of the striatum after nigral injection (17). Interestingly, the combined vector was the most effective. However, these findings have to be cautiously interpreted due to the lack of expression data and the composition of the vector, thus making it difficult to know the exact localization of these proteins in the brain.

CDNF has been studied extensively for Parkinson’s disease since the initial study showed it to be neuroprotective in a 6-OHDA model (2). CDNF was also tested using a mouse model treated with toxin MPTP (1-methyl-4-phenyl-1,2,5,6-tetrahydropyridine), a neurotoxin that has been shown to result in parkinsonism in humans (18). When CDNF was given bilaterally to the striatum before or after the MPTP, the mice showed improved locomotor activity, as well as protection of TH fibers in the striatum and TH^+^ cells in the substantia nigra pars compacta (19). This indicated that CDNF could have a protective effect in toxin models of Parkinson’s disease. Around the same time, another 6-OHDA experiment was performed, in which CDNF was given continuously to the striatum – this also improved rotational behavior, and protection of TH in the striatum and substantia nigra (20). A combination experiment using CDNF and glial cell-derived neurotrophic factor (GDNF) in a 6-OHDA model has been recently performed. However, at the low doses of CDNF used, there was no significant effect on behavior or TH compared to vehicle, although there was indication that CDNF was acting via an ER stress-related mechanism (21). An interesting study used both CDNF alone or in combination with deep brain stimulation after a 6-OHDA lesion of the medial forebrain bundle, a lesion that can be considered severe. When CDNF was injected to the substantia nigra four weeks after the lesion, but not one week, it affected rotational behavior (22). Additionally, when nigral CDNF was combined with deep brain stimulation of the subthalamic nucleus, it affected behavior. It is clear from these studies that CDNF is effective in some cases but not in others. Dose, injection location, lesion paradigm, and single vs chronic infusion, are all important aspects for CDNF to exert its neurotrophic effects in the brain.

In addition to CDNF protein alone, a few studies have used AAV-CDNF in a 6-OHDA model of Parkinson’s disease in rats. AAV2-CDNF administered two weeks before the 6-OHDA toxin improved the rotational behavior, but showed a less robust effect on TH fibers and cells, and only at the highest virus titer (23). This could be due to the spreading of the protein since there was minimal coverage of the striatum in comparison with the protein without viral vector (20,24). Other studies have shown positive results with AAV-CDNF: both AAV2-CDNF (25) and AAV8-CDNF (26) showed positive effects, but the latter was a much milder lesion than in the previous studies.

CDNF was also shown to reduce the number of activated glial cells in the nigra of rats that received a CDNF plasmid carried by a neurotensin-polyplex after a 6-OHDA lesion of the striatum (27). Additionally, CDNF was tested in monkeys and showed an increase in striatal DAT activity after a 6-OHDA lesion (28).

Importantly, a study using cell culture and a large amount of CDNF showed that it was able to protect against α-synuclein preformed fibril-induced cell death (29). This is in accordance with the abovementioned data on MANF.

### Future directions

Both MANF and CDNF have positive effects on models of Parkinson’s disease, however, to determine whether this is through an ER-related mechanism needs further study. Since the clinical trials with neurotrophic factors have shown conflicting results (7,30-32), it remains to be seen whether neurorestorative therapy is feasible. The challenge is that when the disease is diagnosed most of the dopaminergic axons are degenerated, and this seems to be progressing rather fast (33). Nevertheless, what makes MANF and CDNF interesting and distinct from other neurotrophic factors is that intracellular MANF mediates protein homeostasis, but whether this is also the case for extracellularly applied proteins is not yet known. However, currently only CDNF is at the trial stage and only for Parkinson’s disease (34). To make the therapies more effective, one option is to start the neuroprotective therapies earlier. However, for this to be possible we critically need quantitative measures for the disease progression since the Parkinson’s disease rating scale is mostly descriptive and positron emission tomography (PET) ligand studies for dopamine transporter (DAT) are expensive and not available to all. Also, the development of novel drug therapies is most often a linear process where the diagnosis of the disease is not currently connected with it in the best possible way. By combining reporter assays for studying basic biological processes to studies finding biological reporters and disease biomarkers, we can make the drug development not only more rational but also more interconnected with clinically measurable outcomes. These issues are naturally not specific to Parkinson’s disease but present a huge challenge in all neurological diseases.

## Effects in other neurodegenerative diseases

MANF and CDNF have been tested successfully in other models of neurodegenerative diseases. Interestingly, most of the protection seen may be occurring through an apoptotic or inflammatory-related mechanism, rather than the one involving the ER specifically.

MANF is expressed in the human retina and optic nerve (35), and it protects retinal ganglia cells from hypoxia and apoptotic injury *in vitro* and *in vivo* (36). MANF in *D. melanogaster* (DmMANF) was also found to be expressed in the fly visual system neurons and glia, and its removal caused degeneration of glial cells (37). MANF was shown to decrease apoptosis and ameliorate traumatic spinal cord injury in mice (38). Additionally, it was up-regulated in human samples of traumatic brain injury and reduced neuroinflammation in a rat model (39). Interestingly, CDNF was also shown to have a positive effect on spinal cord injury. CDNF was given via bone marrow-derived mesenchymal stem cells in a spinal cord injury model in rats: it had an anti-inflammatory effect, where it protected the cells from injury, and improved locomotor behavior (40). Clearly MANF, as well as CDNF, have some anti-apoptotic and/or anti-inflammatory effects in models that utilize mechanical damage, and MANF is a potential target for retinal diseases.

In addition to these models, both MANF and CDNF have had clear effects in models of Alzheimer’s disease. In cells and a transgenic Alzheimer’s model, MANF was able to protect against cell death caused by amyloid-β, possibly through an ER stress-related mechanism (41). Related to this, CDNF was able to enhance long-term memory in both wild-type and Alzheimer’s transgenic mice (42). However, this study had different outcome measures than the MANF study, and CDNF was not able to increase neurogenesis or decrease amyloid-β build-up.

### Future directions

MANF’s and CDNF’s effects on these models are intriguing and we have some evidence of how they may be occurring. MANF’s role in diabetes has been established using the knockout model (43,44) as well as in patients (45). However, further testing in retinal disease and traumatic injury is needed to understand the mechanisms of MANF’s action as well as its potential therapeutic effects. All of these lines of study may be important for future disease therapies, though in particular these proteins’ effects on both α-synuclein and amyloid-β point to potential use in neurodegenerative disease therapy, a strong unmet need in medicine. As above with Parkinson’s disease, more accurate and earlier diagnostics for synucleinopathies and Alzheimer’s disease are needed for neurotrophic factor therapy so that neuronal projections can be restored before they die. And if these factors can affect abnormal protein build-up in disease, this would be a potentially useful target. However, antibodies to amyloid-β have not fared well in clinical trials (46,47), and it is not fully clear how the aggregation of protein affects disease progression. Although if MANF and CDNF can moderate effects through an anti-inflammatory, ER stress, and/or apoptotic mechanism, this may be more useful for protection than simply aiming to rid the brain of these proteins, particularly if the build-up is in fact a protective mechanism itself. Results from the current clinical trial with CDNF in Parkinson’s disease may provide more information for future potential therapies.

## Effects of MANF and CDNF in stroke

Our previous studies have shown that both recombinant MANF protein and overexpression via AAV vector are neuroprotective when they are applied before cortical ischemia reperfusion injury (3,48). In both cases, the neuroprotection was observed with hastened behavioral recovery over the two-week time period after stroke, indicating not only neuroprotection, but also that MANF has beneficial effects post-stroke. In addition, we found that MANF prevented the apoptosis measured as TUNEL (terminal deoxynucleotidyl transferase (TdT) dUTP Nick-End Labeling) staining when protein was applied directly to the rat brain before distal middle cerebral artery occlusion (3). These results were furthered by our studies in which we administered MANF post-stroke after the lesion had fully developed to study how MANF affects brain repair processes. When MANF was applied via intracerebroventricular injections or infusion, we found that it did not affect the proliferation of neuronal stem cells, but increased the number of neuronal precursors in the areas close to the ischemic lesion (49). These studies also indicated that MANF acts by modulation of proteins of the cell cytoskeleton and initiates an intracellular signaling cascade related to the inflammatory pathways. We found that MANF increased the levels of phosphorylated signal transducer and activator of transcription 3 (STAT3) and particularly serine 727 and tyrosine 705, and that it increased levels of glial-associated intermediate filament (GFAP) in neuronal stem cells (49). Another study in which we used the peri-infarct targeting methods we established (50) showed that post-stroke treatments facilitate functional recovery after stroke. Furthermore, unbiased RNA screening showed that MANF treatment increased the levels of phagocyte markers, indicating that MANF hastens clearance of cell debris after stroke (51). Taken together, these studies indicate that MANF promotes brain recovery after stroke via a mechanism that has both immunomodulatory and neuroregenerative effects in the stroke brain.

The above-mentioned studies were conducted in parallel with the studies on development of the cortex in MANF knockout mice. The studies on cortex development are relevant to discuss here since many cellular processes are similar in development and after ischemic insult in adult brain (eg, activation of glial cells and neurogenesis). In these studies, we found that the developing neurons had shorter neurite extensions as well as slower migration of neurons (52) that correlated with an increase in markers of the unfolded protein response (UPR). Particularly, it is interesting that spliced Xbp1 was increased indicating that activation of the IRE1 pathway of the UPR results in shorter neurite extension and slower cortex development (52). Interestingly, recent studies on the UPR and cortex development indicated that constitutive PERK activity caused inhibition on indirect neurogenesis and smaller cortices (53). Their follow-up study indicated that this can mediate the phenotype of Zika virus infection-caused alterations in brain development (54). Thus, it can be concluded that activation of UPR pathways and chronic ER stress can regulate direction of neurogenesis as well as the dynamics of neurodevelopment.

### Future directions

The questions that remain for future studies are for example, how does MANF modulate the inflammatory cells and which inflammatory cells are involved? Does it affect primarily microglia post-stroke or macrophages, or does it affect other infiltrating immune cells? How does MANF affect the number of phagocytes, and, since they are motile cells, does MANF affect their migration similarly as we observed for developing neurons? In terms of ER stress and different UPR pathways, it is important to study how different pathways affect the changes in neurogenesis and maturation of cells into specific neuronal phenotypes. This can also be extended into regeneration of the adult nervous system using *in vivo* reprogramming as well as transplantation studies with neuroprogenitor cells.

## Effects of extracellularly applied vs endogenous protein

Both MANF and CDNF can function differently depending on whether the role of endogenous protein is measured or whether the proteins are given exogenously to cells or tissue. As expressed proteins, they are localized at the ER lumen and have only minimal secretion from neurons, even after lentiviral overexpression (55). However, it should be noted that a proportion of intracellular and extracellular MANF or CDNF is likely different in non-neuronal cells and needs to be studied separately on each cell type. They both have N-terminal signal sequences, similar to classical neurotrophic factors, which target them to secretion. However, at the C-terminus they have a KDEL retention sequence that retains them from Golgi to the ER lumen (56). Moreover, for MANF it has been shown that the removal of the last seven amino acids, ASARTDL, the retention sequence, causes them to be secreted from neuronal cells (56). However, caution should be used for C-terminal tags since they can interfere with the native expression in the ER. In the lumen, MANF interacts with GRP78 (aka BiP, binding immunoglobulin protein) (57) and it has been recently shown that MANF is involved in the folding of high molecular weight proteins together with GRP78 (58). It was indicated that MANF inhibits ADP release from GRP78 and ATP binding to GRP78, and thereby modulates protein folding in the ER lumen via GRP78. Our result indicates that the CXXC domain at the C-terminus is important for the neuroprotective effect of expressed MANF (59). When they are injected into the brain tissues or when they are applied as recombinant proteins to the extracellular space they act through yet unknown receptor. We know that for MANF the CXXC is important for *in vivo* neuroprotective effects, but not the last seven amino acids (ASARTDL) (59). CDNF was recently shown to be secreted at very low levels compared to MANF from ARPE-19 cells (human retinal pigment epithelial cell line), and therefore that it was mainly in the cell, particularly at the ER (60). Interestingly, when the ER-retention signal of CDNF, KTEL, was deleted, secretion of CDNF was increased, similarly to MANF as mentioned above. When CDNF was injected into the striatum or cortex it readily diffused in the brain tissue and was taken up by neurons in an unspecific manner (24). Electron microscope studies showed that CDNF is found inside endosomes and multivesicular bodies in the brain after recombinant protein injections, but how it mediates its neuroprotective effect needs further investigation. Overall, these findings could indicate different mechanisms depending on if the proteins are applied to the cells or expressed in the cells themselves since it is not fully clear how they may exert their effects.

Evidence for different actions also comes from overexpression studies in cells and protein application *in vivo*. Cultured neurons were protected when MANF was overexpressed, however not when it was applied exogenously (61). Indeed, MANF is drastically different from GDNF since the radiolabeled protein does not bind to the cell surface, indicating that it does not have a classical transmembrane receptor (61). Recently, it has been shown that the N-terminus of MANF binds to sulphatides (62), and this may be how it is internalized after brain parenchymal injections, but this needs to be investigated further as well. Although CDNF has been neuroprotective when given to animals as a protein (in addition to overexpression via viral vector), this does not exclude the possibility that MANF could also be protective in this way. Also, there are other reports of MANF being applied to the culture medium and demonstrating cell protection in non-neuronal cells (44). Perhaps, different cell types respond differently to extracellular vs intracellular MANF, and the brain as opposed to the *in vitro* conditions are vastly different. The latter is unsurprising, however unfortunately we do not have enough evidence to precisely understand how these factors act at the cell surface to exert their protective effects.

What makes MANF interesting is that the chemically-induced UPR increases intracellular MANF levels. Thus, it seems that MANF is able to bypass the translational blockage caused by the UPR, indicating that MANF is involved in coping with the increased levels of unfolded proteins in the ER lumen . Therefore, it may be speculated that similarly to the UPR, an endogenous protective and adaptive pathway for neurons, MANF expression increase is also part of an adaptive and protective pathway for cells. As mentioned above, thapsigargin, tunicamycin, or lactacystin, chemical inducers of the UPR. by blocking the SERCA pump, N-linked glycolysation, or by inhibiting the proteasome, respectively, all increase MANF levels in various cultured cells (63). Our study with the luciferase reporter shows that in cultured SH-SY5Y cells only thapsigargin and ER calcium depletion caused the secretion from cells, and tunicamycin did not (56). Thus, from neurons or neuronal-like cells, MANF and CDNF secretion is not induced by all forms of ER stress, but by ER calcium depletion. For future studies it is interesting to investigate the physiological conditions in which ER calcium is depleted at a magnitude that causes exodosis (departure of ER resident proteins) (64-66).

## Conclusions

CDNF and MANF have been shown to have therapeutic effects in various neurological disease models via administration of recombinant proteins or via the gene therapy approach. Their biology and mechanisms of neuroprotective effects have been challenging to study due to lack of reproducible *in vitro* models. Whether the exogenously added neuroprotective effects are mediated similarly to protein functioning inside the ER lumen still needs to be addressed. The studies conducted so far are very intriguing from the point of view that the endogenous proteins expressed in the ER lumen show therapeutic effects when applied extracellularly. In the future, it will be important to further study the therapeutic potential of ER resident proteins.
